# Hydrothermal-Assisted Sintering Strategy Towards Porous- and Hollow-Structured LiNb_3_O_8_ Anode Material

**DOI:** 10.1186/s11671-017-2234-2

**Published:** 2017-07-25

**Authors:** Haifa Zhai, Hairui Liu, Hongjing Li, Liuyang Zheng, Chunjie Hu, Xiang Zhang, Qiling Li, Jien Yang

**Affiliations:** 1 Henan Key Laboratory of Photovoltaic Materials, College of Physics and Materials Science, Henan Normal University, Xinxiang, 453007 People’s Republic of China; 2 National Laboratory of Solid State Microstructures, Nanjing University, Nanjing, 210093 People’s Republic of China; 3 State Key Laboratory of Chem/Bio-Sensing and Chemometrics, College of Chemistry and Chemical Engineering, Hunan University, Changsha, 410082 People’s Republic of China

**Keywords:** LiNb_3_O_8_, Anode, Lithium-ion batteries, Porous and hollow structure

## Abstract

Porous- and hollow-structured LiNb_3_O_8_ anode material was prepared by a hydrothermal-assisted sintering strategy for the first time. The phase evolution was studied, and the formation mechanism of the porous and hollow structure was proposed. The formation of the unique structure can be attributed to the local existence of liquid phase because of the volatilization of Li element. As the anode material, the initial discharge capacity is 285.1 mAhg^−1^ at 0.1 C, the largest discharge capacity reported so far for LiNb_3_O_8_. Even after 50 cycles, the reversible capacity can still maintain 77.6 mAhg^−1^ at 0.1 C, about 2.5 times of that of LiNb_3_O_8_ samples prepared by traditional solid-state methods. The significant improvement of Li storage capacity can be attributed to the special porous and hollow structure, which provides a high density of active sites and short parallel channels for fast intercalation of Li^+^ ions through the surface.

## Background

In recent years, much attention has been paid to hollow and porous structures due to their widespread applications in catalysis, energy, environmental engineering, drug delivery, and sensor systems [1–4]. Compared with other new energy batteries, lithium-ion batteries (LIBs) have gained commercial success as the predominant power source for portable electronics and show great potential in large-scale applications because of its high energy density, long lifespan, and environmental benignity [5]. To obtain high electrochemical performance, the electrodes of LIBs always have open structures, which can provide a high density of active sites and parallel channels for faster intercalation of Li^+^ ions through the surface [6]. However, it is challenging to synthesize the nanomaterials with open structures including porous and hollow architectures.

To improve LIBs performances, people have been seeking for high-performance electrode materials, including anode and cathode materials. LiFePO_4_ [7], LiCoO_2_ [8], LiMn_2_O_4_ [9], LiVPO_4_F [10], and various hybrid materials [11, 12] have been seriously considered as candidates for cathode materials. For anode materials, many different materials have been investigated as alternatives to graphite-based anode materials, such as transition metal oxides (TMOs) [13], molybdenum disulfide (MoS_2_), and graphene-based hybrids [14]. In recent literature, niobium has been shown to have superior electrochemical performance [15]; some traditional compounds doped with Nb element and novel Nb-based compounds are well developed [16–19]. Nb-based oxides have been considered as promising anode materials for LIBs with improved safety. Compared with Li_4_Ti_5_O_12_ (with a theoretical capacity of 175 mAhg^−1^), Nb-based oxides have a relatively high theoretical capacity of 389 mAhg^−1^. Also, it is notable that the two Nb redox couples, Nb^5+^/Nb^4+^ and Nb^4+^/Nb^3+^, can suppress the formation of solid electrolyte interface (SEI) film during cycling [19]. LiNb_3_O_8_, a well-known material, always appear in the preparation process of LiNbO_3_ as an impurity phase due to Li volatilization [20]. Jian et al. firstly introduced LiNb_3_O_8_ material prepared by a solid-state reaction as an anode for LIBs. It is found that the as-prepared LiNb_3_O_8_ sample ball-milled with acetylene black (LiNb_3_O_8_-BM) largely improved the initial discharge/charge capacities (351 and 212 mAhg^−1^) than those of the as-prepared LiNb_3_O_8_ sample (250 and 170 mAhg^−1^) at 0.05 C; after 50 cycles, the capacity reached 150 mAhg^−1^ for LiNb_3_O_8_-BM at 0.1 C, only 30 mAhg^−1^ for LiNb_3_O_8_ sample [18]. Porous LiNb_3_O_8_ nanofibers also exhibited improved capacity and cyclability in virtue of the high surface area, small nanocrystals, and porous structure with the initial discharge capacity of 241.1 mAhg^−1^ at 0.1 C [19]. Due to the difficulty to obtain pure phase, as a novel anode material with high theoretical capacity, LiNb_3_O_8_ has rarely been studied.

In this paper, porous- and hollow-structured LiNb_3_O_8_ anode material was successfully prepared by a hydrothermal-assisted sintering process. The phase evolution was studied, and the formation mechanism of the porous and hollow structure was proposed. The morphological and electrochemical properties of LiNb_3_O_8_ as the anode material were also studied in detail.

## Methods

### Preparation of Samples

LiNb_3_O_8_ powders were prepared by the hydrothermal-assisted sintering process. Lithium hydroxide monohydrate (LiOH·H_2_O, Aladdin, ACS, ≥98.0%) and niobium pentoxide (Nb_2_O_5_, Aladdin, AR, 99.9%) were purchased as raw materials without further purification. First, 3.5 mmol of Nb_2_O_5_ was dispersed into 35 ml of LiOH·H_2_O transparent aqueous solution (the mole ratio of Li:Nb = 8:1) with magnetic stirring for 1 h. Then, the suspension solution was put into a 50-ml Teflon-lined hydrothermal synthesis autoclave reactor. After that, the reactor was sealed and maintained at 260 °C for 24 h and then cooled down to room temperature naturally. Finally, the as-prepared products were centrifuged and rinsed with deionized water and ethanol. After drying in an oven at 60 °C for 12 h, the white Li-Nb-O powders were collected and calcined at various temperatures from 500 to 800 °C for 2 h with a ramp rate of 5 °C/min.

### Characterization

The thermal decomposition characteristic of Li-Nb-O powder was studied by a thermogravimetric and differential scanning calorimeter (TG/DSC, Netzsch STA 409 PC/PG) from room temperature to 1200 °C with a ramp rate of 10 °C/min under N_2_ atmosphere. The crystal structures of the calcined powders were analyzed using X-ray powder diffraction (XRD; Bruker D8 Discover) with Cu *Kα* radiation. The morphologies of the calcined powders were characterized by scanning electron microscopy (SEM; JSM-6700F). X-ray photoelectron spectroscopy (XPS) analysis was performed on a Thermo-Fisher Escalab 250Xi instrument.

### Electrochemical Measurements

The LiNb_3_O_8_ electrodes were prepared by spreading slurry of LiNb_3_O_8_ powders, carbon black, and polyvinylidene fluoride (PVDF) with a weight ratio of 8:1:1 onto an aluminum foil. Afterward, the electrode was dried at 120 °C in a vacuum oven overnight. The anodes were punched into disks with a diameter of 16 mm. For the electrochemical measurements, CR2025 coin-type cells were assembled in an argon-filled glove box using lithium foil as the counter electrode and polypropylene microporous membrane (Celgard 2320) as a separator to isolate the two electrodes, and then, the 1.0-M LiPF_6_ electrolyte was dissolved in a mixture of ethylene carbonate and dimethyl carbonate (1:1 by volume). Galvanostatic charge-discharge tests of the cells were performed using a Land electric test system (Wuhan Land Electronics Co., Ltd., China) between 0 and 3 V (vs. Li/Li^+^) at different current densities of 0.1–1 C (1 C = 389 mAhg^−1^). Cyclic voltammetry (CV) curves were recorded on an electrochemical workstation (CHI604E, Shanghai Chenhua Instruments Co., Ltd., China) in the voltage range of 1–3 V.

## Results and Discussion

**Fig. 1:**
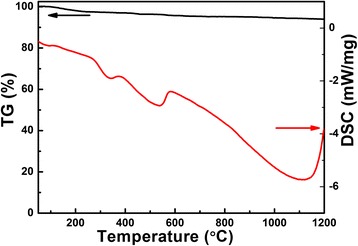
TG/DSC curves of the Li-Nb-O powder from room temperature to 1200 °C at a heating rate of 10 °C/min in N_2_

Figure 1 plots the TG/DSC curves of the powder obtained after hydrothermal reaction without further calcination. The weight loss of the powder is very small, about 5%, even as the temperature reaches to 1100 °C, but the loss is occurring throughout the entire calcination process. This can be attributed to the evaporation of Li element due to its low melting temperature, which is confirmed by the DSC results with endothermic reaction process throughout the entire calcination process. At 330 °C, an endothermic peak occurs, which may originate from the formation of LiNbO_3_. An exothermic reaction occurs at 580 °C resulting from the reaction between LiNbO_3_ and Nb_2_O_5_ to form LiNb_3_O_8_. As seen in the DSC curve, beyond 1100 °C, the exothermic reaction becomes strong due to the decomposition of LiNb_3_O_8_.

**Fig. 2:**
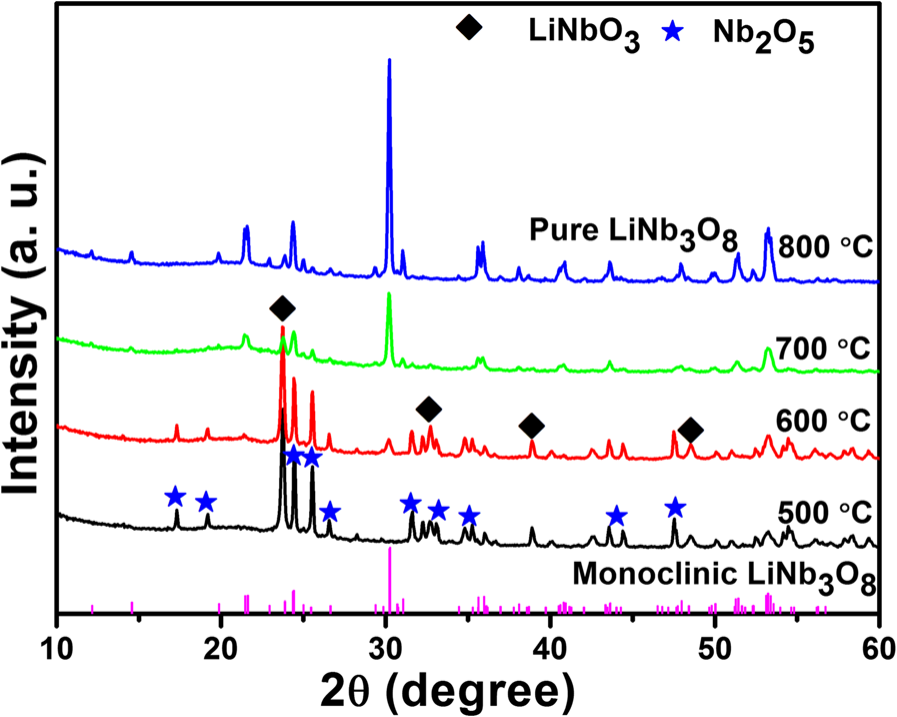
XRD patterns of the Li-Nb-O powder calcined at different temperatures for 2 h

The XRD patterns of Li-Nb-O powders calcined at different temperatures are shown in Fig. 2. It can be seen that the major phases are LiNbO_3_ and Nb_2_O_5_ at 500 °C. With the increase of calcination temperature, the diffraction peak at 30.26° appears which can be indexed as the (410) plane of monoclinic LiNb_3_O_8_. The reaction can be described by Eq. (1) not Eq. (2) [21]:

1 $$ {\mathrm{LiNb}\mathrm{O}}_3+{Nb}_2{\mathrm{O}}_5\to {\mathrm{LiNb}}_3{\mathrm{O}}_8 $$

2 $$ {\mathrm{LiNb}\mathrm{O}}_3\to {\mathrm{LiNb}}_3{\mathrm{O}}_8+{Li}_2\mathrm{O}\uparrow $$

At 700 °C, the monoclinic LiNb_3_O_8_ is the predominant phase with almost negligible impurity. The pure phase of LiNb_3_O_8_ is obtained at 800 °C with all the diffraction peaks indexed to the monoclinic phase (JCPDS card no. 36–0307), a space group of P21/a. Compared with the traditional solid-state method, the pure phase of LiNb_3_O_8_ is more easily obtained using the hydrothermal-assisted sintering process.

**Fig. 3:**
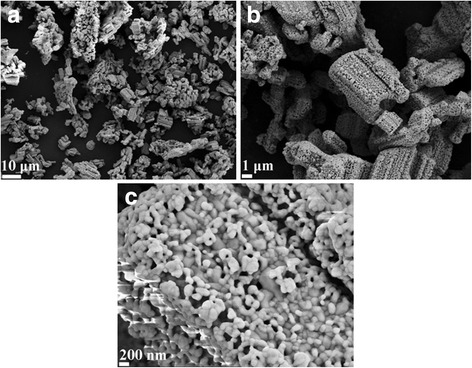
**a**–**c** SEM images of LiNb_3_O_8_ powder with different magnifications

The SEM images of LiNb_3_O_8_ powder calcined at 800 °C with different magnifications are presented in Fig. 3. The porous and hollow structure that resembles a honeycomb is formed by LiNb_3_O_8_ nanoparticles with the length of several micrometers. The structure is not flat, with obvious warping, and even forms closed tubby-like structures. It is fully different from the particle aggregation that results from conventional solid-state reactions. The size of the LiNb_3_O_8_ particle is about 200 nm, as shown in Fig. 3c. The small particle size and unique structure are beneficial to ion intercalation [6]. The formation of the unique structure can be attributed to the lithium volatilization during the calcination process, as proved by TG-DSC results. As the easy volatilization of Li element, the excess Li element existing in the powder easily migrates to the surface of particles and turns into liquid phase. The local existence of liquid phase is conducive to the formation of new LiNb_3_O_8_ particles at the site and also encourages the formation of networks between the particles.

**Fig. 4:**
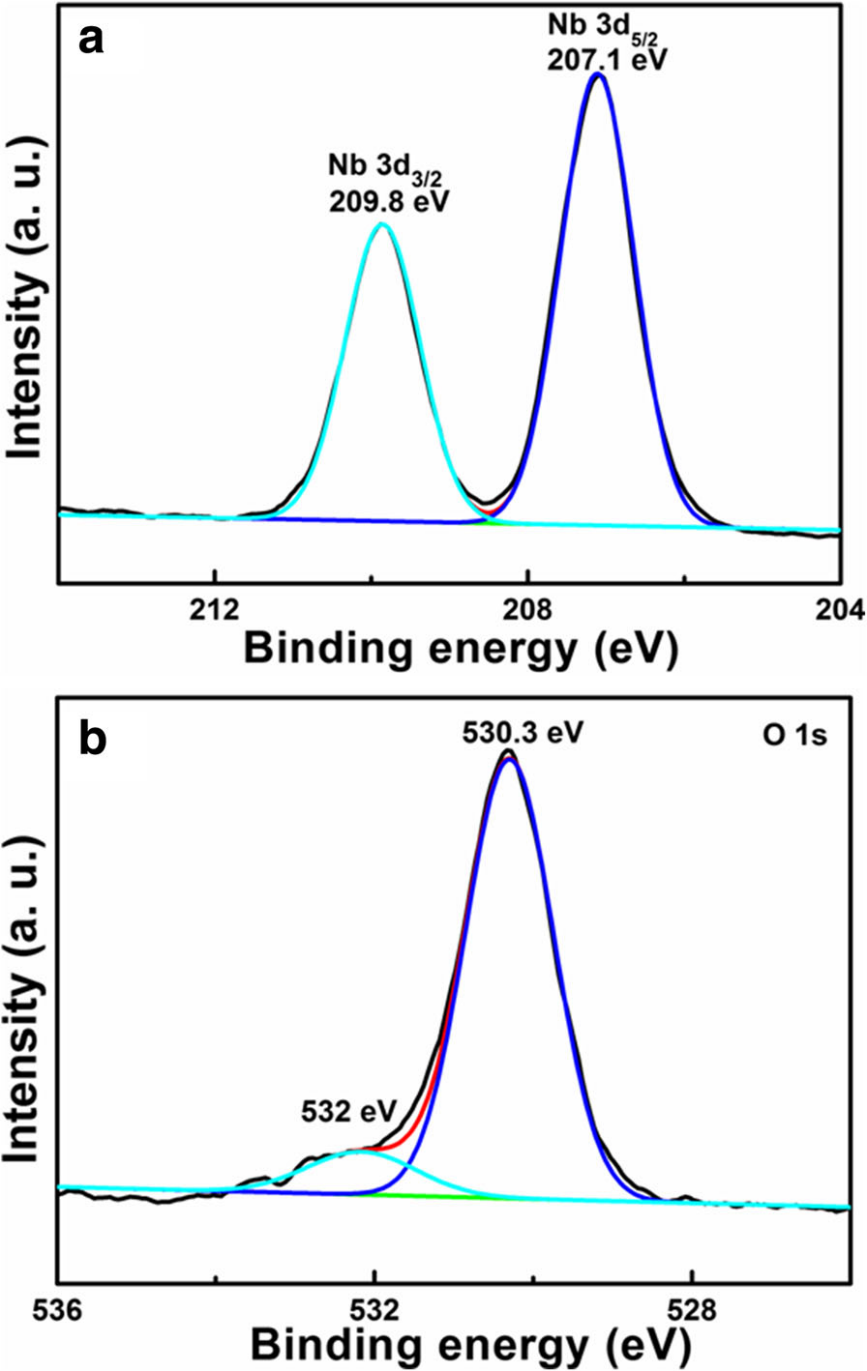
XPS spectra of (**a**) Nb 3d and (**b**) O 1s for the porous- and hollow structured LiNb3O8

To further confirm the elemental compositions and the electronic state, the porous- and hollow-structured LiNb_3_O_8_ powders are analyzed by XPS, as shown in Fig. 4. The XPS data were calibrated using C 1s as a reference with the binding energy at 284.6 eV. In Fig. 4a, two peaks at 207.1 and 209.8 eV correspond to Nb 3d_5/2_ and 3d_3/2_, respectively, indicating the Nb^5+^ state in LiNb_3_O_8_ [22]. The XPS spectra of O 1s in Fig. 4b can be deconvoluted into two peaks at 530.3 and 532 eV. The former is assigned to the Nb-O bonds, and the latter is related to nonlattice oxygen [22, 23].

**Fig. 5:**
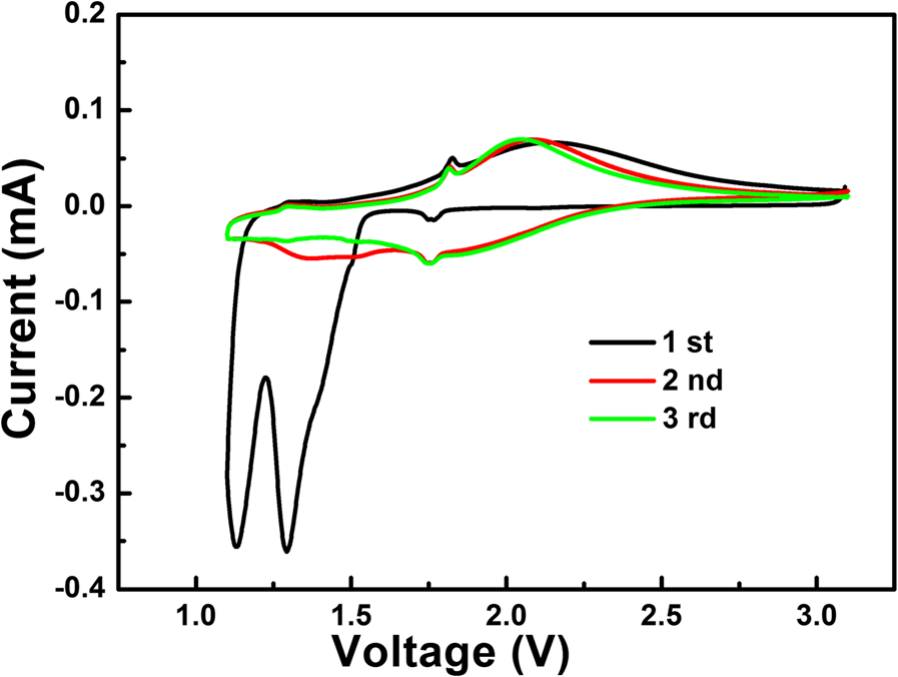
The initial three CV curves of the LiNb_3_O_8_ powder at a scan rate of 0.05 mV/s between the voltage ranges of 3–1 V

To investigate the electrochemical performance of the as-prepared LiNb_3_O_8_ sample, the tests of CV and galvanostatic discharge-charge cycling were performed. The first three CV curves of LiNb_3_O_8_ powder at a scanning rate of 0.1 mV/s in the range of 3~1 V are shown in Fig. 5. In the first cycle, two pronounced peaks (Li insertion) are observed at 1.13 and 1.30 V; the former can be attributed to the partial reduction of Nb^4+^ to Nb^3+^, while the latter can be related to the full valence variation of Nb^5+^ to Nb^4+^ [18, 19]. As seen in Fig. 5, the subsequent cycles are quite different from the first cycle. The disappearance of the peaks at 1.13 and 1.30 V implies the phase transition in the first cycle is irreversible. Only the oxidation (Li extraction) peaks at 1.71 and 1.96 V remain stable upon cycling, implying the structure change of the LiNb_3_O_8_ sample in subsequent cycles is reversible.

**Fig. 6:**
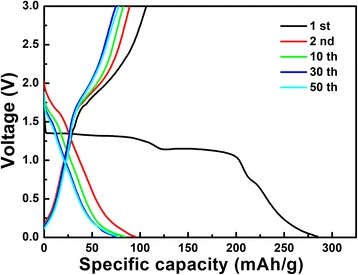
Galvanostatic charge-discharge profiles of the LiNb_3_O_8_ powder at 0.1 C between 3 and 1 V

Figure 6 shows the discharge-charge curves of the LiNb_3_O_8_ powder at 0.1 C (here, 1 C = 389 mAhg^−1^) between 3 and 1 V in the first, second, tenth, thirtieth, and fiftieth cycles. In the first discharge curve, two obvious potential plateaus can be observed at approximately 1.13 and 1.30 V, which is in good agreement with the CV results that two phase reactions, Nb^4+^ → Nb^3+^ and Nb^5+^ → Nb^4+^, take place. However, in the subsequent cycles, the plateaus that exist in the first cycle are replaced by sloping curves, implying different reactions between the first and subsequent cycles. At the same time, the initial discharge capacity of the LiNb_3_O_8_ sample is 285.1 mAhg^−1^ at 0.1 C, the largest discharge capacity reported so far for LiNb_3_O_8_ anode materials [18, 19]. 4.4 Li per unit formula can be inserted into the LiNb_3_O_8_ material, corresponding to a composition of Li_5.4_Nb_3_O_8_. However, the charge capacity in the first cycle is 106.4 mAhg^−1^, indicating that only 1.6 Li can be extracted reversibly. The big loss of 2.8 Li is ambiguous at present.

**Fig. 7:**
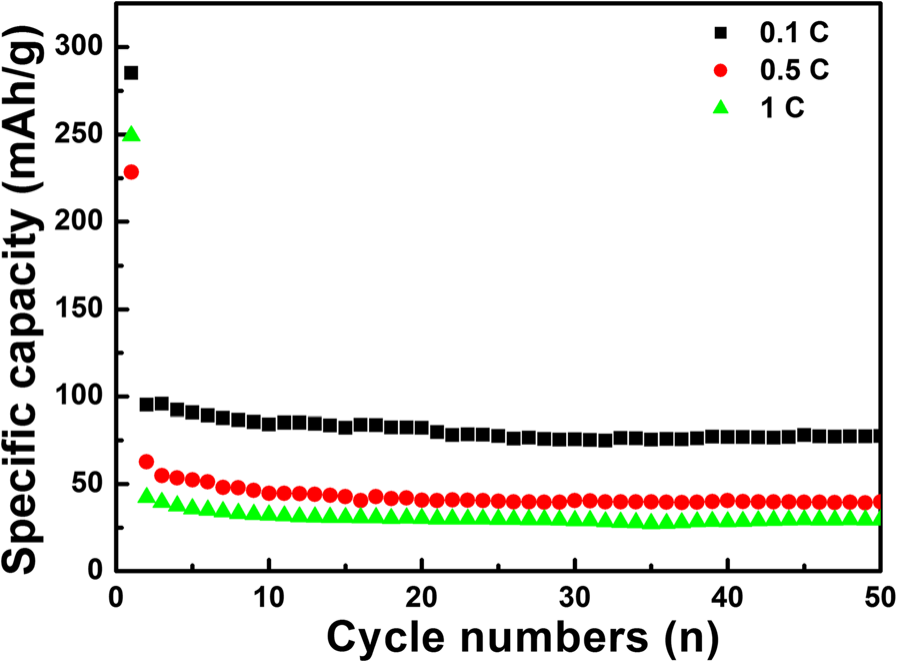
Cycling performance of the LiNb_3_O_8_ powder at different current rates of 0.1 C, 0.5 C and 1 C

Figure 7 shows the cycling performance of LiNb_3_O_8_ sample at different current rates up to 50 cycles. The initial discharge capacities of LiNb_3_O_8_ powder at rates of 0.1, 0.5, and 1 C are 285.1, 250, and 228 mAhg^−1^, respectively. At a current rate of 0.1 C, the reversible capacity can still maintain 77.6 mAhg^−1^, about 2.5 times of that of LiNb_3_O_8_ samples prepared by traditional solid-state method (about 30 mAhg^−1^ at 0.1 C, Ref. [18]). The significant improvement of Li storage capacity can be attributed to the special porous and hollow structure of LiNb_3_O_8_ sample, which provides a high density of active sites and short parallel channels for faster intercalation of Li^+^ ions through the surface [6]. When the rates increase to 0.5 and 1 C, the discharge capacities after 50 cycles remain 39.7 and 29.4 mAhg^−1^, respectively. It is expected that the capacity stability can be improved by suitable surface modification on LiNb_3_O_8_ material.

## Conclusions

In summary, porous- and hollow-structured LiNb_3_O_8_ anode material was successfully prepared by the hydrothermal-assisted sintering strategy. The phase evolution was studied, and the formation mechanism of the porous and hollow structure was proposed. The formation of the unique structure can be attributed to the local existence of liquid phase because of the Li volatilization. As the anode material, the initial discharge capacity is 285.1 mAhg^−1^ at 0.1 C, the largest discharge capacity reported so far for LiNb_3_O_8_. After 50 cycles, the reversible capacity can still maintain 77.6 mAhg^−1^, about 2.5 times of that of the LiNb_3_O_8_ samples prepared by traditional solid-state methods. The significant improvement of Li storage capacity can be attributed to the special porous and hollow structure of LiNb_3_O_8_ powder, which provides a high density of active sites and short parallel channels for fast intercalation of Li^+^ ions through the surface.
